# Inverse effects of tDCS over the left versus right DLPC on emotional processing: A pupillometry study

**DOI:** 10.1371/journal.pone.0218327

**Published:** 2019-06-19

**Authors:** Jens Allaert, Alvaro Sanchez-Lopez, Rudi De Raedt, Chris Baeken, Marie-Anne Vanderhasselt

**Affiliations:** 1 Department of Head and Skin, Ghent University, University Hospital Ghent (UZ Ghent), Department of Psychiatry and Medical Psychology, Ghent, Belgium; 2 Ghent Experimental Psychiatry (GHEP) Lab, Ghent University, Ghent, Belgium; 3 Department of Experimental Clinical and Health Psychology, Ghent University, Ghent, Belgium; 4 Department of Clinical Psychology, Universidad Complutense de Madrid, Madrid, Spain; 5 Department of Psychiatry, Vrije Universiteit Brussel (VUB), University Hospital UZ Brussel, Brussels, Belgium; University Medical Center Goettingen, GERMANY

## Abstract

**Background and objectives:**

The Dorsolateral prefrontal cortex (DLPFC) is implicated in cognitive and emotional responses. Yet, research that investigates the causal role of the left versus right DLPFC during the processes of emotion appraisal is lacking. In the current study, transcranial direct current stimulation (tDCS) was used to disentangle the functional lateralization of the DLPFC on emotional processing in response to the anticipation of, and subsequent confrontation with emotional stimuli in healthy volunteers.

**Methods:**

Forty-eight subjects received both active and sham (on separate days) anodal tDCS over either the left (N = 24) or right (N = 24) DLPFC. Subjects’ pupil dilation (PD, a physiological marker of cognitive resource allocation) was recorded while performing an appraisal task in which negative and positive emotion eliciting images were presented, each preceded by an informative cue indicating the valence of the upcoming stimulus.

**Results:**

As compared to sham stimulation, left DLPFC anodal tDCS resulted in increased PD when confronted with negative emotional images, whereas right DLPFC anodal tDCS resulted in decreased PD when confronted with emotional images, irrespective of valence.

**Limitations:**

The interpretation of pupil dilation in response to emotional stimuli is limited.

**Conclusion:**

These findings suggest inverse lateralized DLPFC effects on cognitive resource allocation (as measured by pupillary responses) when confronted with emotional stimuli. The current findings may shed some light on mechanisms that explain the antidepressant effects of non-invasive brain stimulation of the left DLPFC.

## Introduction

The regulation of emotional responses is essential for subjective wellbeing [1], and plays an important part in dealing with daily life hassles. Prefrontal cortico-limbic networks including the dorsolateral prefrontal cortex (DLPFC) and the ventrolateral prefrontal cortex (VLPFC) play an important role in this modulation of emotional responses [2–6]. For instance, it has been shown that when individuals are instructed to down-regulate their emotional responses towards emotional stimuli (i.e., reappraisal), activity in the DLPFC increases, while activity in the amygdala and self-reported negative affect decreases [5]. It is well known that the DLPFC has lateralized functioning. Increased right DLPFC activity is associated with emotion/mood dysregulation and deficits in the regulation of emotional attention [7], whereas increased left DLPFC activity is related to efficient emotion and emotional attention regulation [8]. In the same context, patients with mood disorders are characterized by a functional hemispheric imbalance of the DLPFC, with hyperactivity of the right DLPFC and hypoactivity of the left DLPFC [9]. Moreover, by using non-invasive brain stimulation (NIBS) techniques, augmenting activity in the left DLPFC or reducing activity in the right DLPFC has been shown to decrease depressive symptoms (for a review, see [10]). Research in healthy volunteers showed that anodal (i.e., excitatory) tDCS over the left DLPFC decreased emotional reactivity towards negative stimuli, as measured by both behavioral [11, 12], and psychophysiological [13] assessment methods.

To date, even though NIBS is optimal to study lateralization of DLPFC in emotional processing, research in healthy populations is currently limited to the scope of selective emotional attention [14]. Specifically, it has been shown that anodal (i.e., excitatory) tDCS over the right DLPFC leads to diminished emotional attentional disengagement, whereas anodal tDCS over the left DLPFC leads to improved emotional attentional disengagement. These latter effects were observed in a task requiring participants to intentionally implement top-down attention regulation (i.e., divert gaze away from emotional stimuli when prompted to engage with their neutral counterparts; [14, 15]). In other words, participants had to follow instructions in order to perform the emotional attention task. Yet, the DLPFC is implicated in the maintenance and implementation of goal-oriented behaviour (e.g., cognitive control, selective attention, [16]), and these lateralized effects might be related to attentional set differences between left and right DLPF activity [17–19]. Moreover, the DLPFC is also involved in other anticipatory (related to attentional set) and reactive cognitive processes (e.g., [20]), that also play a role in emotional processing [21]. Emotional responses arise both when confronted with an emotion-eliciting event, as well as when anticipating such an event. For instance, the anticipation of a stressful event has been shown to generate similar emotional and physiological responses as the stressful event itself [22, 23], and anticipatory emotional processes may play an important role in emotion and stress regulation [24, 25]. Research is therefore warranted to investigate the functional lateralization of DLPFC functioning on emotional processing, when anticipating emotional stimuli and responding naturally (i.e., absence of task instructions) to these. Such research is important as it resembles real life contexts more closely (i.e., absence of explicit task instructions and/or pre-existing performance goals) and may shed light on the outcomes of a functional hemispheric DLPFC imbalance on emotional processing, thereby contributing to neuropsychological depression research.

Taken together, the aim of the current study was to investigate—using anodal tDCS versus sham (placebo stimulation)—the functional lateralization of the DLPFC (and its associated prefrontal cortico-limbic network) in regard to emotional processing when individuals respond naturally to emotional stimuli (i.e., response phase), and where they receive time to prepare to deal with these emotion-eliciting stimuli (i.e., anticipation phase), without explicit instructions on how to respond to such stimuli. For this purpose, pupil dilation was measured during the anticipation and response phase. Pupil dilation (i.e., an increase in pupil size), caused by the iris dilator muscle, is under control of the sympathetic nervous system [26, 27] and has been shown (under constant luminance conditions) to be positively related to emotional arousal [28–30], cognitive load, mental effort, conflict processing, [31–34], and emotion regulatory effort [35–39]. Taken together, pupil dilation seems to reflect cognitive resource allocation for the processing of (emotional) stimuli, and can be measured during the anticipation of, and confrontation with, emotion-eliciting stimuli [25, 40].

First, based on previous research demonstrating reduced emotional reactivity towards negative stimuli after anodal tDCS applied to the left DLPFC (suggested due to an increase of cognitive control; [3, 11–13]), we hypothesized that anodal tDCS applied to the left DLPFC (compared to sham tDCS) would result in larger PD (i.e., increased allocation of cognitive resources for emotional processing) during the presentation of negative stimuli (H1). Second, based on previous studies in which excitatory NIBS applied to the right DLPFC produced acute deficits in selective emotional attention [14, 15, 41], which in turn may contribute to impairments in emotion regulatory processes [42, 43], we hypothesized that anodal tDCS applied to the right DLPFC (versus sham tDCS over the right DLPFC) would result in decreased PD (i.e., decreased allocation of cognitive resources for emotional processing) during the presentation of emotional stimuli (H2). Finally, in view of an absence of research in which the causal role of the left and right DLPFC on emotional anticipatory processes during natural responding (without task instructions) is investigated, we have no clear surrounding hypothesis, and this section remains exploratory (but an innovative part of our study).

## Materials and methods

### Participants

Forty-eight healthy participants (aged 18–26; *M* = 21.35; *SD* = 2.21; 67% female) participated in the study. A post-hoc power analysis was conducted with G*Power [44] to compute the observed power, using the observed group × stimulation × phase × valence effect size (ηp2 = .12) and sample size (N = 48) as input, showing power (1- β) = .98. Participants were recruited through Facebook and the Ghent University web service. Selection criteria for participation was: right handedness, normal or corrected to normal vision (only contact lenses, glasses were not allowed due to technical issues with the eye-tracker to collect data from these participants), no pregnancy at time of stimulation, no history or current neurological or psychiatric disorders (checked using the Dutch version of the MINI screening; [45, 46]), no current use of psychoactive substances or psychotropic medication, no history of serious head injury. The experiment was conducted in accordance with the Declaration of Helsinki and the approval of Ghent University’s Medical Ethical Committee was obtained in advance. Furthermore, all participants provided written informed consent at the start of the experiment. Participants received € 30 for participation.

### Materials

#### Emotional appraisal paradigm

**Fig 1:**
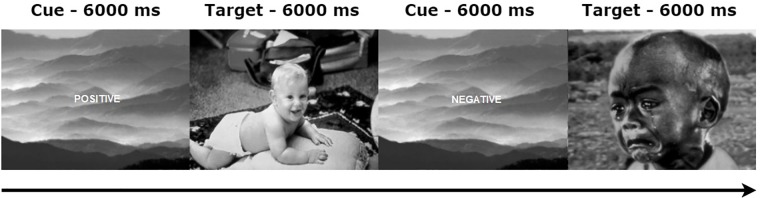
Emotional appraisal task.

Participants were presented with a set of 30 positive (i.e., pleasant) and 30 negative (i.e., unpleasant) gray scaled pictures selected from the International Affective Picture System (IAPS; [47]). The following IAPS pictures were used for negative emotions: 1019, 1205, 1300, 2117, 2800, 2811, 2911, 3170, 3530, 3550, 6021, 6212, 6213, 6250, 6550, 6560, 6570, 8230, 8430, 8480, 9040, 9181, 9250, 9252, 9254, 9300, 9423, 9571, 9594, and 9635. For positive emotions, the following IAPS pictures were used: 1340, 1441, 1659, 1710, 1920, 2040, 2045, 2151, 2158, 2306, 2314, 2332, 2339, 2340, 2352, 2373, 2392, 2395, 2550, 4542, 4599, 4625, 4641, 5829, 5831, 5833, 5836, 7325, 8350, and 8540. Based on IAPS normative data, the perceived valence (on a 1 to Likert 9 scale, where 9 reflects completely positive and 1 reflects completely negative) of positive (*M* = 7.35; *SD* = .57) and negative (*M* = 2.52; *SD* = .70) pictures differed significantly, *t*(58) = 29.25, *p* < .001. Moreover, the arousal ratings (on a 1 to 9 Likert scale, where 9 reflects completely aroused and 1 reflects completely unaroused) between positive (*M* = 4.70; *SD* = .59) and negative (*M* = 6.16; *SD* = .52) pictures also differed significantly, *t*(58) = 10.41, *p* < .001. Each picture was displayed for 6 seconds, and a cue (e.g., the word ‘negative’ or ‘positive’), was presented during the 6 seconds prior to each picture’s appearance. The cue always indicated the correct valence of the upcoming picture, thereby informing subjects whether the upcoming stimulus would elicit negative or positive emotions. The order of the picture presentation was pseudo randomized in advance, with the constraint that no more than three pictures of the same valence were presented consecutively. Moreover, pictures were arranged in 3 blocks of each 20 trials, in which the frequency of both picture valences was equal in each block. Participants were told that a series of pictures would be displayed on the screen, and that a cue preceding each picture would inform them about its valence. Participants were instructed to respond naturally to each picture and to look at it for the whole time it was displayed on the screen. In order to control for confounding effects of luminance on pupil dilation [28], the grey-scaled pictures of both valence types were corrected to have similar luminance values. There was no significant difference in luminance between positive (*M* = 90.35; *SD* = 30.88) and negative (*M* = 104.38; *SD* = 23.61) pictures, *p* > .1. Stimulus presentation was programmed in MATLAB and stimuli were displayed on a 22-inch Mitsubishi 2070SB CRT monitor, using a Cambridge Research System ViSaGe visual stimulus generator. The stimulus generator featured a contrast resolution of 14 bits per gun. The average luminance of the monitor was 104 cd/m2 (4002 Td) and the output of the display was gamma corrected. Subjects were seated approximately 75 cm from the monitor, using a fixed head rest. See Fig 1 for a visualization of the paradigm.

#### Transcranial direct current stimulation (tDCS)

**Fig 2:**
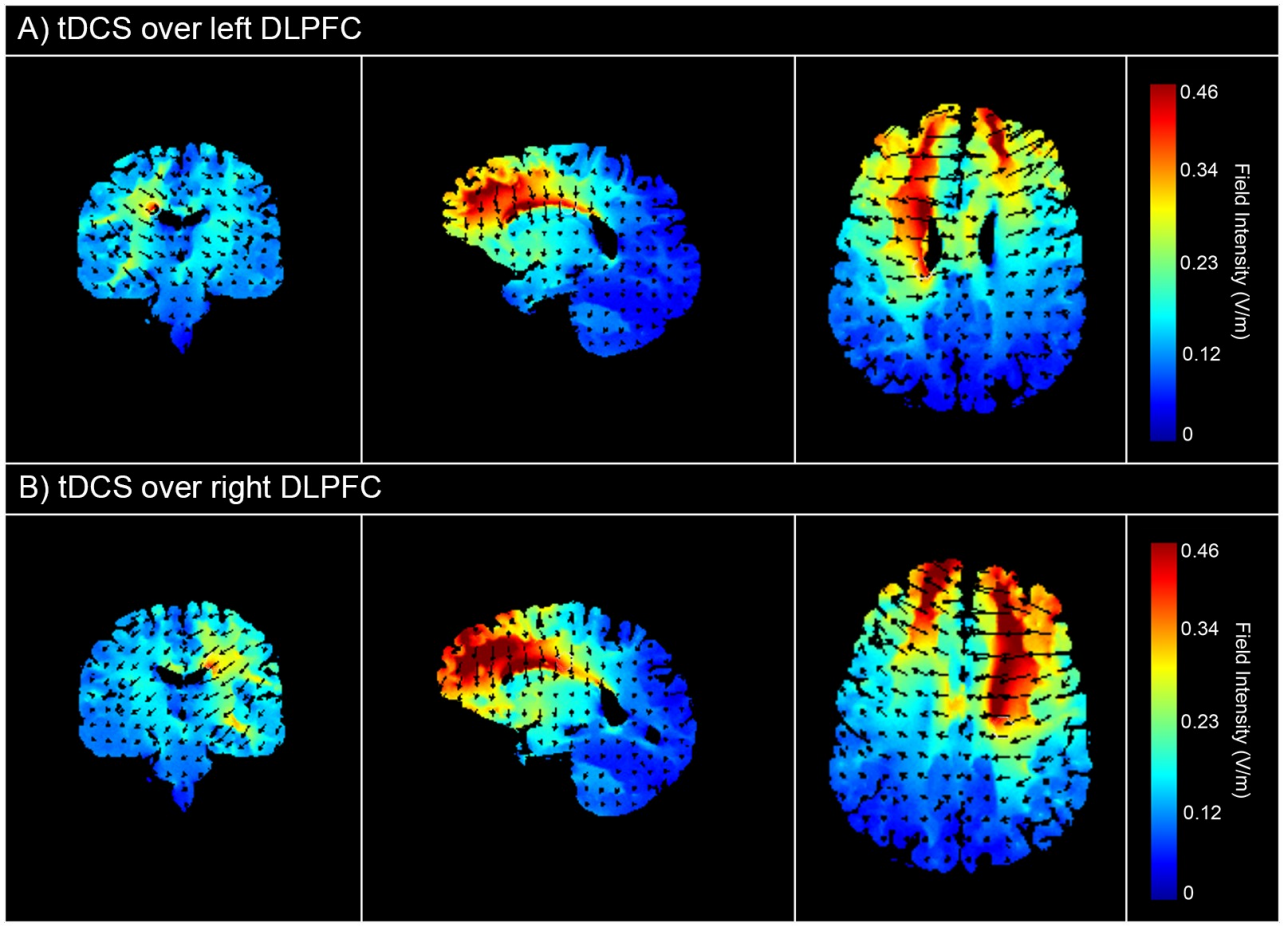
Electric field simulations for tDCS over the left and right DLPFC. The scale represents the electric field (V/m) induced by the anode and cathode.

TDCS was applied with a saline-soaked pair of surface sponge electrodes (35 cm2) and delivered with a battery-driven stimulator (DC-Stimulator Plus, neuroConn GmbH). TDCS modulates the membrane potential of neurons through either depolarization (anodal tDCS) or hyperpolarization (cathodal tDCS), thereby reducing the neuron threshold and increasing neuronal excitability (excitatory effect) or increasing the neuron threshold and reducing the neuronal excitability (inhibitory effect), respectively [48]. To stimulate the left DLPFC, the anode electrode was vertically positioned over F3 according to the 10–20 international system for electroencephalogram electrode placement, whereas the right DLPFC was stimulated by positioning the anode electrode vertically over F4. The cathodal reference electrode was horizontally positioned over the supra-orbital area, contralateral to the anodal electrode location (Fp2 and Fp1, respectively). The electrode placement and method of localization is in accordance with previous tDCS studies, and has shown to produce significant effects on cognitive and emotional processing in a vast range of studies [48, 49]. A constant, direct current of 2 mA (current density = .06), with 15 seconds of ramp up and ramp down, was applied for 20 minutes. For sham stimulation (i.e., placebo stimulation condition in each group) the electrode placement was identical, however the current was directly ramped down after the initial ramp up phase. This procedure entails a reliable sham procedure because subjects are not able to discriminate real from sham stimulation [48]. Fig 2 shows a visualization of the electric field simulation for the left DLPFC (Fig 2A) and right DLPFC (Fig 2B) tDCS montage, which was performed using Soterix HD-Explore software.

#### Eye tracker

Pupillary responses were recorded during the appraisal task using a Cambridge Research System High-Speed Video Eye tracker (CRS HS-VET) at a sampling rate of 250 Hz, resulting in 1500 data samples for each phase (i.e., cue and target) of the trial. The eye tracker and CRT monitor were controlled using the CRS MATLAB toolboxes, and the eye tracking data was transferred to a computer along with event markers indicating the start and valence of every trial, along with the respective phases (cue, target). At the beginning of the task, the eye-tracker was calibrated by instructing subjects to direct their gaze towards 9 dots that were randomly presented on the screen, one by one. Dots were displayed in the four corners of the display, midway between each corner, and in the middle of screen.

#### Questionnaires

To evaluate temporary changes in mood and physical states from before (T_pre_) to after (T_post)_ stimulation, ratings were obtained, using six visual analog scales (VAS; [50]). The six VASs comprised 3 mood measures (i.e., happiness, anger, sadness) and 3 measures of physical states (i.e., fatigue, vigor, tension). Participants were asked to describe how they felt at that moment, by indicating on horizontal 100 cm lines to what extent they experienced each state, ranging from “totally not” to “very much”.

### Procedure

**Fig 3:**
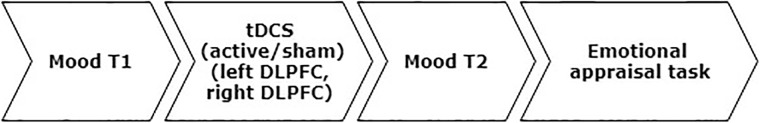
Session protocol.

Participants were randomly assigned to either the left or right DLPFC stimulation group. A single-blind randomized crossover within-subjects design was used for each group: each participant received both active and sham tDCS (either over the left or right DLPFC) on two separate days, with an interval of at least 2 days (*M* = 8.67 days, *SD* = 3.43 days, range: 2–16 days). The order of both stimulation sessions (active and sham tDCS) was counterbalanced across participants. At the start of the experiment, after providing written informed consent, participants reported their mood on the self-report visual analog scales. Next, participants underwent either active or sham tDCS over either the left or right DLPFC, for a period of 20 minutes. All participants were exposed to the same stimulation context in both stimulation sessions, by asking them to remain calmly seated, to not talk or read, until the stimulation was finished. After the stimulation, participants again reported their mood on the visual analogue scales. Subsequently, participants performed the appraisal paradigm, which lasted for roughly 12 minutes. Finally, participants were debriefed and paid € 30 for their participation. For a visualization of the session procedure, see Fig 3. This study was part of a larger project investigating attentional control. During the experimental protocol, before performing the appraisal task, participants performed an attentional engagement-disengagement task and completed several questionnaires (e.g., emotion regulation strategies, rumination, and anxiety). These results are reported by Sanchez-Lopez et al. [14].

### Pupil data preprocessing

First, the pupil data were visually inspected. A consistent trend in pupil dilation was observed during each first trial of every block, compared to consecutive trials. As pupil dilation is sensitive to changes in luminance, and the screen before each first trial of every block is relatively dark compared to the subsequent brighter grey-scale screens during trials, we argued that these systematic differences were due to difference in luminance. Therefore, the pupil data of every first trial from every block were excluded from subsequent analysis, resulting in 27 positive trials and 30 negative trials. Next, invalid data points containing eye blinks, missing or invalid data were linearly interpolated using the interp1 function in Matlab. Next, the data were smoothed, by means of a moving average, using the fastsmooth function in Matlab, with a 5-frame width and a 2-pass filter. Then, the data were detrended (to remove slow irrelevant changes) within each block with simple linear regressions, using the detrend function in Matlab. Consequently, a baseline correction was applied by subtracting the average pupil diameter during the first 165 ms of each trial from the pupil diameter on the consecutive data points during the trial. These pre-processing steps and baseline correction timespan are consistent with prior studies using similar designs [25, 51]. Then, the pupil data from all trials for every subject was averaged, per condition, excluding trials for which 50% or more contained invalid data. This resulted in four pupil waveforms (cue-positive, cue-negative, target-positive, and target-negative) for every subject. Across all conditions, all trials were retained in 79% of the subjects. Finally, in accordance with previous research [28], the mean pupil response for each condition was calculated in a window from 2 to 6 s after picture or cue onset, thereby excluding the initial pupil light reflex (0 to 2s).

### Data analytic plan

First, to assess whether tDCS affected reported mood and physical states, two mixed MANOVA’s were conducted, with one featuring the mood states as dependent variables and the other featuring the physical states as dependent variables. These models included *group* (left DLPFC, right DLPFC) as between-subject factor, *stimulation* (active tDCS, sham tDCS) and *time* (pre-stimulation, post-stimulation) as within-subject factors.

Second, to investigate whether tDCS over the left versus right (relative to sham) differentially affected pupillary responses, a mixed ANOVA with *group* (left DLPFC, right DLPFC) as between-subject factor, *stimulation* (active tDCS, sham tDCS), *phase* (cue, target) and *valence* (negative, positive) as within-subject factors, and pupillary changes as dependent variable, was conducted. Then, to investigate the specific effect of tDCS over the left and right DLPFC, two repeated measures ANOVA’s were conducted for each group (left DLPFC, right DLPFC) separately, with *stimulation* (active tDCS, sham tDCS), *phase* (cue, target) and *valence* (negative, positive) as within-subject factors, and pupillary responses as dependent variable. For the left DLPFC, to address hypothesis 1, a planned comparison (paired t-test) was conducted, testing the difference in pupillary responses during negative targets between active and sham stimulation. For the right DLPFC, to address hypothesis 2, a planned comparisons (paired t-test) was conducted, testing the difference in pupillary responses during the target phase between active and sham stimulation. Furthermore, observed interaction effects that were outside of the scope of our hypotheses, were decomposed using Bonferroni-corrected pairwise comparisons, whereas planned comparisons where not corrected for multiple comparisons, as these did not involve multiple (exploratory) pairwise comparisons and were hypothesis-driven [52]. Additionally, the potential presence of non-significant tDCS effects during the cue phase were followed-up with Bayesian paired samples t-tests.

## Results

### Mood and physical states

Both the mixed MANOVA with the mood states as dependent variables, as well as the mixed MANOVA with the physical states as dependent variables, both with *group* (left DLPFC, right DLPFC) as between-subject factor, *stimulation* (active tDCS, sham tDCS) and *time* (pre-stimulation, post-stimulation) as within-subject factors, yielded non-significant effects of *group* × *stimulation* × *time*, all *F’s* < .39, all *p’s* > .76, or *stimulation* × *time*, *F’s* < 1.36, all *p’s* > .27, suggesting that tDCS did not influence mood or physical states. Table 1 displays descriptive statistics regarding mood.

**Table 1:** Descriptive statistics regarding mood.

Variables	Left DLPFC (N = 24)		Right DLPFC (N = 24)	
	Active tDCS	Sham tDCS	Active tDCS	Sham tDCS
	*M (SD)*	*M (SD)*	*M (SD)*	*M (SD)*
Fatigue (T_pre_)	27.71 (16.70)	33.08 (20.70)	26.58 (16.05)	31.13 (21.54)
Fatigue (T_post_)	36.92 (22.90)	38.17 (20.58)	49.00 (30.59)	44.08 (22.56)
Vigor (T_pre_)	58.63 (14.10)	59.34 (17.70)	56.50 (23.86)	58.54 (21.29)
Vigor (T_post_)	56.58 (18.11)	55.50 (14.23)	43.58 (23.54)	50.00 (21.47)
Anger (T_pre_)	5.96 (9.54)	7.00 (9.74)	5.17 (6.81)	5.63 (7.70)
Anger (T_post_)	5.67 (8.80)	7.33 (7.39)	4.08 (6.70)	7.75 (12.13)
Tension (T_pre_)	19.17 (20.96)	14.86 (15.11)	11.29 (11.71)	16.29 (18.28)
Tension (T_post_)	16.42 (16.39)	13.29 (11.46)	17.96 (24.68)	20.79 (22.51)
Sadness (T_pre_)	9.75 (13.02)	8.88 (10.41)	7.17 (7.97)	6.46 (7.44)
Sadness (T_post_)	7.25 (9.87)	6.96 (7.94)	7.08 (11.10)	5.63 (7.42)
Happiness (T_pre)_	62.88 (12.97)	63.92 (15.87)	66.46 (23.33)	69.58 (20.79)
Happiness (T_post)_	62.38 (14.06)	60.96 (12.84)	58.67 (20.53)	58.75 (19.80)

### tDCS effects on pupillary changes

Table 2 displays descriptive statistics regarding PD for the various conditions. A mixed ANOVA with *group* (left DLPFC, right DLPFC) as between-subject factor, *stimulation* (active tDCS, sham tDCS), *phase* (cue, target) and *valence* (negative, positive) as within-subject factors, was conducted. An effect of *phase* was observed, *F*(1, 46) = 253.48, *p* < .001, *η*_p_^2^ = .85, with higher pupillary changes in the target phase (*M* = .11, *SD* = .01), compared to the cue phase (*M* = -.19, *SD* = .01). In addition, a main effect of *valence* was observed, *F*(1, 46) = 20.132, *p* < .001, *η*_p_^2^ = .30, with higher pupillary changes during negative trials (*M* = -.01, *SD* = .01), compared to positive trials (*M* = -.072, *SD* = .01). However, these *phase* and *valence* effects were accounted by a *phase* × *valence* effect, *F*(1, 46) = 8.86, *p* = .01, *η*_p_^2^ = .16. Bonferroni-corrected pairwise comparisons showed that the effect of *phase* was significant for both negative, *t*(47) = 12.53, *p* < .001, *d* = 1.81, and positive trials, *t*(47) = 13.97, *p* < .001, *d* = 2.02, whereas the effect of *valence* was only significant during the cue phase, *t*(47) = 4.81, *p* < .001, *d* = .70, but not during the target phase, *t*(47) = 1.28, *p* = .82. Furthermore, a significant effect of *group* × *stimulation* × *phase*, *F*(1, 46) = 4.84, *p* = .03, *η*_p_^2^ = .10, and a significant effect of *group* × *stimulation* × *phase* × *valence*, *F*(1, 46) = 5.98, *p* = .02, *η*_p_^2^ = .12, was observed. All remaining effects (i.e., *group*, *stimulation*, *group* × *stimulation*, *group* × *phase*, *group* × *valence*, *stimulation* × *phase*, *stimulation* × *valence*, *group* × *stimulation* × *valence*, *group* × *phase* × *valence*, *stimulation* × *phase* ×*valence*) were non-significant (all *Fs* < 2.11, all *p*s > .15). Further analyses including session order (i.e., active tDCS in the first session or active tDCS in the second session) as a between-subject factor in the mixed ANOVA showed that both the stimulation × phase × group, F(1, 44) = 4.25, p = .04, ηp2 = .09, and the stimulation × phase × valence × group, F(1, 44) = 4.67, p = .04, ηp2 = .09, effects remained significant, indicating the effect was not dependent on session order. Order was not implied in any main or interaction effects. To test the hypothesized inverse tDCS effects over the left and right DLPFC on pupillary changes, the four-way *stimulation* × *phase* × *valence* × *group* interaction was followed up by testing the *stimulation* × *phase* × *valence* interaction separately for each group.

**Table 2:** Descriptive statistics regarding pupil dilation changes relative to baseline.

		Left DLPFC (N = 24)		Right DLPFC (N = 24)	
Phase	Valence	Active tDCS	Sham tDCS	Active tDCS	Sham tDCS
		*M* (*SD*)	*M* (*SD*)	*M* (*SD*)	*M* (*SD*)
Cue phase	Negative	-.17 (.16)	-.14 (.16)	-.10 (.15)	-.17 (.13)
	Positive	-.25 (.15)	-.27 (.13)	-.22 (.13)	-.22 (.15)
Target phase	Negative	.15 (.13)	.07 (.11)	.10 (.09)	.14 (.09)
	Positive	.08 (.09)	.12 (.12)	.07 (.11)	.12 (.10)

### Effects of tDCS over the left DLPFC on pupillary changes

**Fig 4:**
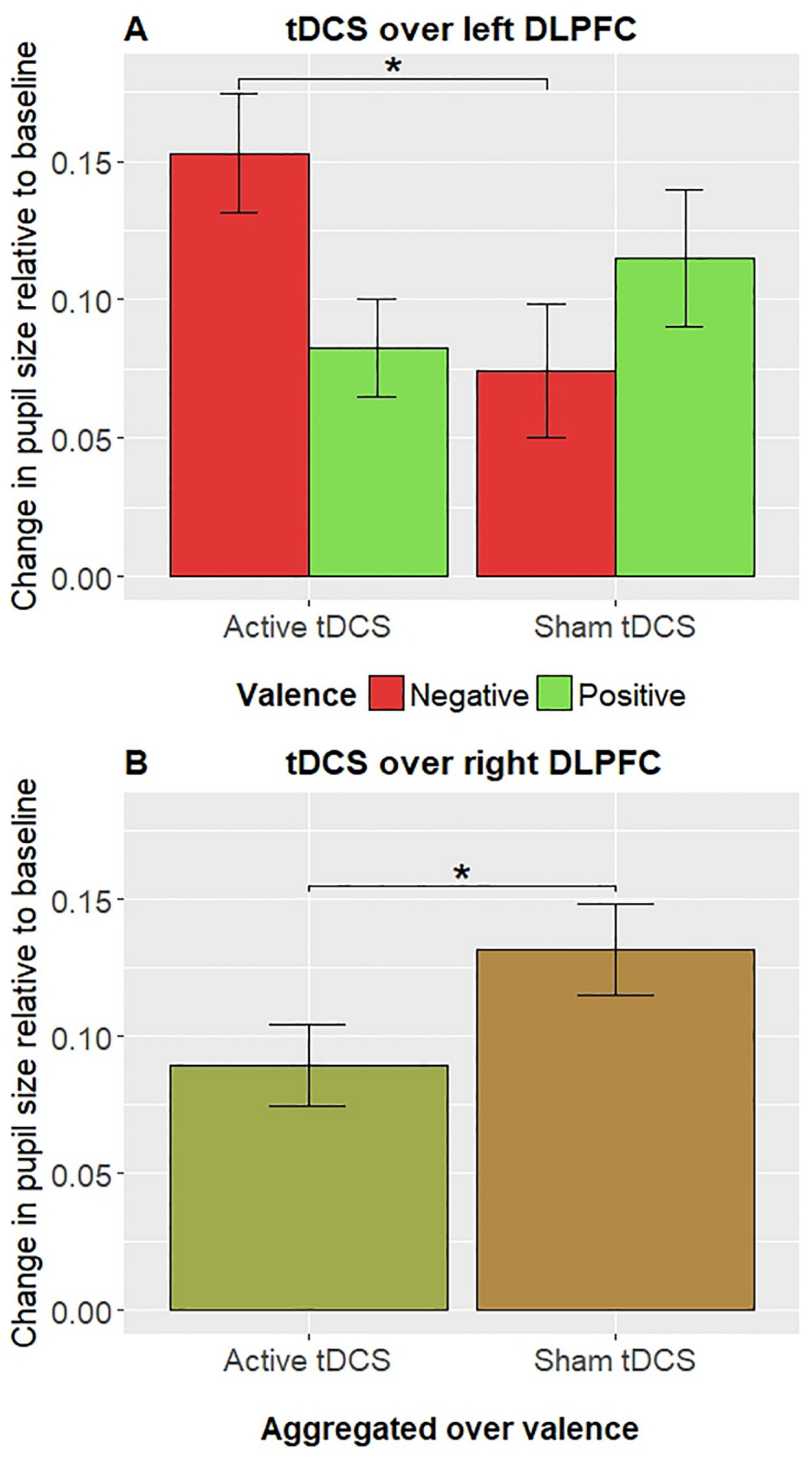
TDCS effects of left (A) and right (B) DLPFC on pupil dilation during the target phase.

A repeated measures ANOVA with *stimulation* (active tDCS, sham tDCS), *phase* (cue, target) and *valence* (negative, positive) showed a significant *stimulation* × *phase* × *valence* interaction, *F*(1, 23) = 4.98, *p* = .04, *η*_p_^2^ = .18. The planned comparison showed a significant difference in pupillary responses between negative targets during active tDCS, compared to sham tDCS, *t*(23) = 2.53, *p* = .02, *d* = .52 (see Fig 4A). Specifically, when viewing negative targets, pupil dilation was larger during active tDCS over the left DLPFC (*M* = .15, *SD* = .13), compared to sham tDCS over the left DLPFC (*M* = .07, *SD* = .11). No significant difference between active and sham tDCS was observed in pupillary responses during the cue phase of negative trials, *t*(23) = .82, *p* = .42, or in the cue phase of positive trials, *t*(23) = .61, *p* = .56. Furthermore, post-hoc Bayesian paired samples t-tests were conducted to investigate the relative support for H_O_ (i.e., there is no difference between active and sham tDCS) versus H_1_ (i.e., there is a difference between active and sham tDCS over the left DLPFC) on pupillary responses during the cue phase. For positive cue’s, H_O_ was 4.66 times more likely than H_1_, BF_01_ = 4.66, whereas for negative cue’s, H_O_ was 1.46 times more likely than H_1_, BF_01_ = 1.46. Taken together, there is anecdotal to moderate evidence for H_O_ (i.e., there is no difference in pupillary responses during the cue phase between active and sham tDCS over the left DLPFC).

### Effects of tDCS over the right DLPFC on pupillary changes

A repeated measures ANOVA with *stimulation* (active tDCS, sham tDCS), *phase* (cue, target), and *valence* (negative, positive), showed no significant *stimulation* × *phase* × *valence* interaction, *F*(1, 23) = 1.26, *p* = .27, *η*_p_^2^ = .05, however, a significant two-way *stimulation* × *phase* interaction was observed, *F*(1, 23) = 5.17, *p* = .03, *η*_p_^2^ = .18. The planned comparison showed a significant difference in pupillary responses between targets during active tDCS, compared to sham tDCS, *t*(23) = 2.25, *p* = .04, *d* = .47 (see Fig 4B). Specifically, when viewing targets (irrespective of valence), pupillary changes were smaller during active tDCS over the right DLPFC (*M* = .09, *SD* = .08), compared to sham tDCS over the right DLPFC (*M* = .13, *SD* = .07). Moreover, in the cue phase, no significant difference between active and sham tDCS was observed in pupillary responses, *t*(23) = 1.44, *p* = .16. A post-hoc Bayesian paired samples t-test was conducted to investigate the relative support for H_O_ (i.e., there is no difference between active and sham tDCS over the right DLPFC) versus H_1_ (i.e., there is a difference between active and sham tDCS) on pupillary responses during the cue phase. H_O_ was 1.88 times more likely than H_1_, BF_01_ = 1.88, indicating anecdotal to moderate evidence for H_O_ (i.e., there is no difference in pupillary responses during the cue phase between active and sham tDCS over the right DLPFC).

## Discussion

The aim of the current neuro-modulation study was to investigate the functional lateralization of the DLPFC (and its associated prefrontal cortico-limbic network) in regard to cognitive resource allocation (as indexed by pupil dilation) for emotional processing when naturally responding during the anticipation of, and confrontation with, emotional stimuli. Essentially, both groups (left and right sham controlled tDCS) were comparable on depressive symptoms and mood, suggesting neither pre-existing nor task related differences in emotional processing. Moreover, results suggested no differential effects of tDCS versus sham stimulation on mood or physical states, which would be consistent with previous research showing that among healthy individuals, NIBS over the DLPFC is able to influence emotional processing, without specifically modulating mood [53].

Overall, our results support the assumption of differential effects of tDCS applied to the left versus the right DLPFC in pupillary responses to emotion-eliciting stimuli. First, anodal versus sham tDCS applied to the left DLPFC resulted in increased PD when attending to negative, but not positive images. Pupil dilation has been associated with both emotional arousal [28], cognitive effort [54], and also activity in the DLPFC [37]. Other authors argued that pupil dilation reflects top-down regulation of emotional stimuli (e.g., [55, 56]). In the current study, participants received no instructions to regulate or use effort; they were simply asked to respond naturally and spontaneously to every emotion-eliciting stimulus (participants carefully looked at the pictures, and did not close their eyes). Therefore, in the current study, although pupil dilation does not indicate about intentional regulation effort, it does inform about automatic changes in emotional processing (i.e., cognitive resource allocation) due to neuromodulation of the DLPFC. Furthermore, it informs about the change in the sympathetic nervous (and arousal related neurochemical) system following the identification and appraisal of an emotion-eliciting stimulus. Furthermore, it is probable that the increased cognitive resource allocation (due to active tDCS over the left DLPFC), contributes to improved (emotion) regulatory top-down processes (i.e., cognitive control). This would be in line with research suggesting that improved cognitive control is an important working mechanism in the effects of NIBS over the left DLPFC in reducing depressive symptoms [8, 10, 57], and emotional reactivity [11–13]. However, this is speculative and further research is required to specifically investigate whether these changes in resource allocation contribute to improved cognitive control and reduced emotional reactivity.

Second, anodal versus sham tDCS over the right DLPFC resulted in decreased PD when confronted with emotional stimuli, irrespective of their emotional valence. The data show a difference in dilation due to real versus placebo stimulation, denoting an adjustment in sympathetic nervous system in response to tDCS. Prior studies using excitatory NIBS over the right DLPFC showed adverse effects on emotional attention (e.g., increased attention towards negative stimuli, reduced attentional control over emotional information; [14, 41, 58–60]), which in turn is suggested to contribute to maladaptive emotion regulatory processes [42, 61]. These findings would be consistent with the notion that excitatory right DLPFC stimulation contributes to decreased cognitive resource allocation to process emotional information, impeding individuals’ capacity to engage in emotion regulatory processes. As mentioned earlier, a functional hemispheric imbalance of the DLPFC is a hallmark of depression, with increased activity in the right DLPFC relative to the left DLPFC [9]. Moreover, this increased activity in the right DLPFC is suggested to contribute to depressive symptoms, such as increased negative mood [7]. In addition, depressed individuals typically display impaired performance on experimental paradigms in which they have to exert cognitive effort to inhibit emotional information [62–64]. Altogether, results of the current study add to this literature by showing that PD decreases following tDCS over the right DLPFC, possibly denoting a reduction in cognitive resource allocation to process emotional information. Of importance, in contrast to the findings of the left DLPFC, this effect was independent of emotional valence. The origin of this valence right DLPFC non-specific effect in contrast to the valence specific left DLPFC is puzzling, and is in line with previous inconsistent results produced by previous research (for a review, see [53]). For instance, the results of one study showed increased attention towards negative stimuli after excitatory NIBS over the right DLPFC [41], whereas another showed reduced attentional control towards emotional information (irrespective of valence; [14]). Thus, further research is required to investigate in which specific contexts valence specific effects are produced.

Third, effects of tDCS during the anticipation phase were non-significant. Furthermore, post-hoc Bayesian paired t-tests for both the left and right DLPFC showed anecdotal to moderate evidence for the absence of tDCS effects. Conceptually, it could be that the current paradigm did not incentivize participants to proactively engage in emotional regulatory processes during the anticipation phase, as the stimuli have no personal relevance. Future studies could use more self-relevant and emotionally provoking stimuli, such as (rigged) social feedback, as the need to belong is a fundamental human motivation and social feedback automatically prompts self-regulatory processes [65–67].

Besides a number of strengths, such as a comparison of the left versus right DLPFC, and the employment of an appraisal paradigm allowing natural responding, it has to be noted that the current study has several limitations. First, only pupillary responses were measured. As a result, it is difficult to interpret what pupil dilation reflects, given that PD has been shown to reflect both arousal and cognitive effort to control emotional responses [28, 29]. Future studies would benefit from including additional, complementary psychophysiological and behavioral measures, such as skin conductance response (an index of arousal; [68, 69]), changes in heart rate variability (an index of emotional regulation success; [70]), and self-report ratings of arousal and valence. Second, based on the normative IAPS data, positive and negative images not only differed on valence, but also on arousal (negative images being more arousing than positive images). However, this does not affect the validity of the conclusions of the current study, as inferences were restricted to the comparison between real and sham stimulation, and not between negative and positive stimuli. Third, tDCS produces diffuse effects, resulting in not only the neuro-modulation of the target area (DLPFC), but also of its underling neuronal network and neighboring brain regions [71–73]. For instance, the VLPFC is also implicated in emotional processing, and is located near the DLPFC [5]. Furthermore, research has shown that anodal tDCS over the VLPFC affects emotional processing and emotional reactivity [74–77]. Therefore, some caution is warranted for the specificity of the DLPFC stimulation in the current study. Future studies would benefit from the inclusion of neuro-imaging assessment, which would allow to investigate the specific affected neuro-circuitry.

To conclude, the results of the current study corroborate the hypothesis of inverse lateralized DLPFC functioning on cognitive resource allocation for emotional processing when naturally responding to emotional stimuli and extend previous findings of inverse lateralized DLPFC effects on emotional attention processes [14]. This study established a basic proof of concept for inverse lateralized DLPFC effects on resource allocation for emotional processing (as measured by pupillary responses) when attending anticipated emotional stimuli. The current findings may shed some light on the outcomes related to right DLPFC hyperactivity observed in depression, and on how left DLPFC tDCS may produce antidepressant effects.
